# Early Spatio-Temporal and Cognitive Deficits in Alzheimer’s Disease

**DOI:** 10.3390/jcm14020579

**Published:** 2025-01-17

**Authors:** Tina Iachini, Mariachiara Rapuano, Francesco Ruotolo, Alessandro Iavarone, Sabrina Iuliano, Gennaro Ruggiero

**Affiliations:** 1 Department of Psychology, Università degli Studi della Campania “L. Vanvitelli”, 81100 Caserta, Italy; santa.iachini@unicampania.it (T.I.); mariachiara.rapuano@unicampania.it (M.R.); francesco.ruotolo@unicampania.it (F.R.); sabrina.iuliano@unicampania.it (S.I.); 2 Laboratory of Clinical Neuropsychology, Neurological Unit of “Ospedali dei Colli”, 80131 Naples, Italy; aleiavarone@gmail.com

**Keywords:** egocentric/allocentric spatial reference frames, temporal order, normal aging, Alzheimer’s disease

## Abstract

**Background/Objectives:** Mental representation of spatial information relies on egocentric (body-based) and allocentric (environment-based) frames of reference. Research showed that spatial memory deteriorates as Alzheimer’s disease (AD) progresses and that allocentric spatial memory is among the earliest impaired areas. Most studies have been conducted in static situations despite the dynamic nature of real-world spatial processing. Thus, this raises the question: Does temporal order affect spatial memory? The present study, by adopting a dynamic spatial memory task, explored how the temporal order of item presentation influences egocentric and allocentric spatial judgments in individuals with early-stage Alzheimer’s disease (eAD) and healthy elderly individuals (normal controls—NC). **Method:** Participants were required to memorize dyads of simple 3D geometrical objects presented one at a time on a desk along with a bar. Afterwards, they had to choose what stimulus appeared either closest to them (egocentric judgment) or closest to the bar (allocentric judgment). **Results**: Results revealed that the temporal order significantly affected spatial judgments in eAD patients but not in NC participants. While eAD patients remain anchored to the item presented first, which is more accurate regardless of the frame used, NC are equally accurate with the item that appears first or second. This is presumably because eAD patients struggle to flexibly shift attention and update spatial representations in dynamic situations, which leads to reliance on initial information and difficulties with information presented later. **Conclusions:** This highlights the importance of further understanding the cognitive strategies employed by AD patients.

## 1. Introduction

Alzheimer’s disease (AD) is a neurodegenerative disorder that progressively disrupts cognitive and behavioral functions [1,2]. It primarily affects the hippocampus-entorhinal cortex (HP-EC) network, which is critical for spatial and episodic memory [3]. Early in AD, amyloid-beta Aβ plaques accumulate in the HP-EC, affecting neurons crucial for spatial navigation and memory [4]. As a result, early AD patients often experience spatial disorientation and wandering, especially in navigating familiar environments, serving as early indicators of the disease (e.g., [5,6,7]).

These symptoms, present in about 60% of early-stage AD (eAD) patients, highlight the HP-EC’s role in spatial navigation [4]. A degradation of the ability to use environmental cues for navigation shifts reliance from allocentric to egocentric strategies. Egocentric frames use the body for spatial localization, relying on self-referential cues; allocentric frames utilize external landmarks and are hippocampus-dependent, enabling mental map formation independent of current position [8,9,10,11,12,13,14,15]. The shift arises because the allocentric strategy, heavily dependent on the hippocampus, becomes challenging as these structures degenerate early in AD [10,16,17].

Research consistently shows spatial memory deterioration as AD progresses. Notably, allocentric spatial memory is among the earliest impaired areas, as demonstrated in fMRI studies reporting that the brain regions associated with allocentric encoding (e.g., hippocampus, medial entorhinal cortex) are less active in AD patients than in control groups during spatial tasks. This leads to poor allocentric but not egocentric performance, as the latter is related to areas outside the hippocampus, which may be less affected in the eeAD [17,18].

Studies using Virtual Reality (VR) reveal that AD patients struggle to replicate or navigate previously learned routes, a sign of impaired spatial memory [19,20,21]. Moreover, while healthy controls can flexibly switch between allocentric and egocentric strategies based on environmental demands, AD patients predominantly use egocentric cues even when allocentric navigation would be more efficient [6,22].

Overall, these findings underscore that given the allocentric disruption, egocentric strategies become predominant, although they too decline as AD progresses [23]. Moreover, AD patients also exhibit lower flexibility in switching between reference frames. A behavioral study requiring participants to alternate between spatial representations has demonstrated that eAD patients have lower flexibility in adapting between these perspectives, particularly when switching from an allocentric to an egocentric frame [24]. This difficulty in switching frames of reference suggests a broader disruption in the cognitive processes that manage spatial representations, likely tied to HP-EC network degeneration [24]. Furthermore, a study compared eAD patients, patients with amnestic variant of Mild Cognitive Impairment (aMCI), and normal controls (NC) on the capacity to provide categorical or abstract (i.e., right/left) and coordinate or metric (i.e., distance-based) judgments according to egocentric or allocentric frames of reference. aMCI and eAD patients, as compared to NC, showed selective deficits of coordinate allocentric judgments with categorical allocentric judgments being spared [25]. Therefore, the inability of the elderly to represent metric distances between spatial elements could be another possible marker to detect the transition from healthy aging to AD.

Prior research focusing on egocentric and allocentric spatial memory has mainly used static spatial layouts. In real-world scenarios, however, individuals frequently process spatial information in a dynamic, sequential manner—such as arranging objects on a table in a specific order or navigating through a room while encountering various items in succession. This raises the question: Does temporal order affect spatial memory?

In a recent study, Iachini and colleagues [26] addressed this issue by recruiting a sample of healthy young adults. A dynamic version of the Ego-Allo spatial memory task was devised [27]. Participants had to memorize the position of two geometric 3D objects, positioned at different distances from them and an external bar. Crucially, the objects were dynamically presented one after the other. The task was to judge which object appeared closest to them (egocentric target) or to the bar (allocentric target). Therefore, the target object could be the first or the second in both cases. The authors found that egocentric performance improved when the object closest to the body appeared first, while allocentric performance improved when the object closest to the bar appeared second. Thus, a flexible influence of temporal order on spatial representations in healthy young people emerged, presumably linked to the flexible attentional shift towards the target frame (body or bar).

During aging, individuals typically experience various cognitive and neurophysiological changes that can impact their ability to process spatial information dynamically. For example, older adults may show a decline in cognitive flexibility and attentional resources, which are crucial for integrating dynamic spatial information in real-time contexts [28,29]. AD patients face more significant challenges due to the progress of degeneration that compromises the flexible switch between egocentric and allocentric frames, especially in scenarios where temporal order plays a critical role in decision-making. Thus, examining the performance of healthy elderly and AD patients in dynamic spatial tasks is essential for understanding the impact of aging and neurodegeneration on spatial representation.

To this aim, we adopted the same dynamic spatial memory task [26] to compare patients with early AD diagnosis (eAD) and healthy adults (NC), matched by age and education. Moreover, we assessed four cognitive functions that could be closely linked to egocentric and allocentric spatial processes: visual attention and planning through the Trail Making Test (TMT [30]); visuo-spatial working memory through the Corsi Block Tapping Test Forward and Backward [31,32,33]; perceptual discrimination of stimuli with the Sgorbi Test [33]; and the ability to monitor actions and inhibit irrelevant responses with the Frontal Assessment Battery (FAB; [34,35]).

On the basis of previous literature, we hypothesized that egocentric and allocentric performance should be worse in eAD patients than in NC. Moreover, if eAD are less flexible than NC, then they should keep anchored on the first target object in any case. In addition, we hypothesized an association between attentional-executive and visuo-spatial capacities on the one hand and egocentric and allocentric processing capacities on the other. Finally, to exclude the possibility that the results were due to a general limitation of visual discrimination, we also analyzed perceptual judgments about the dimension of stimuli, without expecting significant differences between eAD and NC.

## 2. Materials and Methods

### 2.1. Participants

Fourteen eAD patients (9 males; age range: 60–82 y, M = 71.36 y, SD = 6.91; education years M = 10.43 y, SD = 4.31) were selected for the study at the Ospedale dei Colli Aminei, C.T.O (Napoli, Italy). Twenty-eight NC (14 males; age range: 62–81 y, M = 70.36 y, SD = 4.80; education years M = 10.89, SD = 5.24) were recruited from senior citizen centers in the city of Naples (Italy). The NC group matched eAD patients in regard to both age and education. All participants volunteered to take part in the experiment and gave their written informed consent. Moreover, they were right-handed and had normal or corrected-to-normal vision. Recruitment and testing were carried out in accordance with the local Ethics Committee requirements and the 2013 Helsinki Declaration [36].

As regards the eAD group, patients fulfilled the NINCDS-ADRDA criteria for AD [37]. The mean MMSE score was 20.86, SD = 3.46 (corrected = 21.40, SD = 3.33). NC group had a mean score at MMSE of 27.18, SD = 2.05 (corrected score = 27.40, SD = 1.80). The two groups did not differ significantly in age (F < 1) and years of education (F < 1).

### 2.2. Experimental Sessions

The study consisted of two sessions. In the first session, participants underwent the four neuropsychological tests, that is, the Frontal Assessment Battery (FAB [34,35]), Trail Making Test (TMT [30]), Sgorbi Test (SGORBI [33]), and Corsi Block Tapping Test (CORSI [31,32,33]). In the second session, participants underwent the dynamic version of the Ego-Allo Task [26]. The whole testing was performed in a soundproof and comfortable room.

#### 2.2.1. Session 1: Neuropsychological Assessment

The FAB, TMT, SGORBI, and CORSI Tests were given to all participants.

The Frontal Assessment Battery (FAB [34,35]) is a brief cognitive and behavioral test for the assessment of executive functions. It comprises six subtests that explore several capabilities such as conceptualization, mental flexibility, motor programming, sensitivity to interference (e.g., handling conflicting instructions), inhibitory control, and environmental autonomy (e.g., grasp reflex). The performance is measured by mean accuracy.

The Trail Making Test (TMT [30]) is a test for the assessment of visual attention (part A) and task switching (part B) and is particularly useful for detecting dementia-related cognitive disorders. In this experiment, we administered only part A, which deals with visual-spatial detection ability, number recognition, visual-motor coordination, and tracking speed. Participants were required to connect numbers from 1 to 25 as fast as possible; the score was the time taken (in seconds) to complete the task.

The Sgorbi Test (SGORBI [33]) assesses the capacity to visually discriminate stimuli. The test consists of 32 pairs of spiral-shaped stimuli. Out of these, 16 pairs are identical and 16 are different. Subjects had to make a judgment of the type same/different. Performance is scored by accuracy and response time.

The Corsi Block Tapping Test (CORSI) evaluates visual-spatial working memory capacity [31,32,33]. It takes the form of a wooden board with nine blocks presenting numbers from 1 to 9 on the side of the experimenter. The experimenter hints a sequence of blocks, and the participant must replicate the sequence at once. The test begins with a two-block sequence, increasing in complexity. To proceed, participants need to accurately replicate at least two out of three trials for each length of the sequence; otherwise, the test is interrupted, and span ability is recorded. In this study, we used both the forward and backward versions of block tapping. In the forward version, participants replicated the sequence in the same order as the experimenter. In the backward version, participants reproduced the sequence in reverse order, starting from the last block tapped. In both conditions, mean accuracy measured the performance.

#### 2.2.2. Session 2: Spatio-Temporal Ego-Allo/Task

##### Materials

We used a dynamic version of the static “Ego-Allo Task [26]. The dynamic features of the task were obtained by manipulating two variables: space and time. Specifically, here we manipulated the temporal order in which egocentric and allocentric targets were presented. The participants had to provide egocentric and allocentric spatial judgments (“Which object was closest to you/the bar?”, respectively). The correct stimulus (the target) could have appeared first or second.

##### Setting and Stimuli

The virtual-reality scenario was built by means of the 3D Vizard Virtual Reality Software Toolkit 5 (Worldviz, LLC, Santa Barbara, CA, USA) and presented using the same software on a computer screen positioned around 50 cm from the participants seated in a comfortable chair.

The stimuli were created using the program Sketchup Pro 2018, a 3D modeling software, based on those used by Iachini and colleagues in previous studies (e.g., [25,26,28]. The set included four easily recognizable geometric objects (cube, pyramid, sphere, and cone) different in color (shades of gray) and size (big objects = 8 × 8 cm; small objects = 6 × 6 cm). These objects were shown in pairs (i.e., dyads) on a virtual table (50 × 35 × 2 cm) that also featured a black bar on one side.

**Figure 1:**
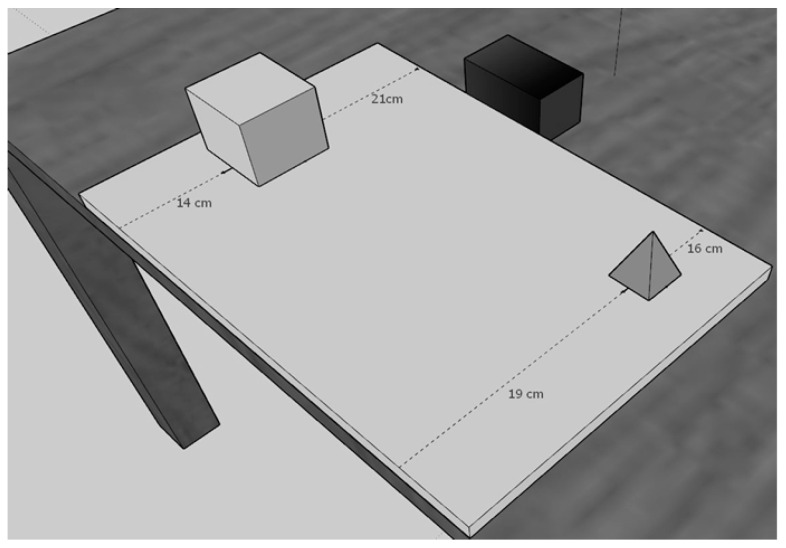
The figure depicts the position of the 3D geometrical objects (e.g., cube and pyramid) on the panel such that in each dyad the metric difficulty for allocentric and egocentric judgments was the same. In this case, the egocentric metric difficulty based on the distance of both the cube and pyramid from the participant’s body was 5 cm (i.e., 19 cm–14 cm), and the allocentric metric difficulty based on the distance of both the cube and pyramid from the Black Bar was also 5 cm (i.e., 21 cm–16 cm).

The objects could be positioned on the table at different distances from the observer and the black bar. The different distances between the two objects and the reference frame determined the metric difficulty of the dyad for egocentric and allocentric judgments. For example, if an object was positioned 8 cm and the other object 13 cm away from the participant, the difference (13 − 8 = 5) represented the metric difficulty for the egocentric judgment. As regards the allocentric judgement, if an object was 22 cm and the other object was 17 cm away from the black bar (i.e., allocentric target), once again the difficulty was 5 cm (22 − 17 = 5). Three levels of metric difficulties were devised: easy = 11 cm; medium = 8 cm; difficult = 5 cm (see Figure 1).

Based on these constraints (i.e., the same metric difficulty for egocentric/allocentric judgments in each dyad), 80 dyads were devised: 40 dyads comprising cube and pyramid and 40 dyads comprising sphere and cone. The 80 dyads were organized into four blocks, each of which corresponded to a specific object pair (Cube–Pyramid or Cone–Sphere) and a specific spatial judgment (egocentric or allocentric). Each block comprised 20 trials requiring 16 spatial judgments and 4 perceptual judgments. The latter served as distractors and to assess simple visual discrimination ability.

### 2.3. Procedure

Before the experimental session, participants gave their written consent to take part in the study. As regards eAD patients, written consent was obtained from both the patients and the caregiver in charge after detailed clarification of the purpose and methods of the study.

Then, participants were given verbal and written instructions about the experimental task. They had to memorize the spatial positions and dimensional features (size) of the two stimuli, presented one at a time, and then to judge which of the two stimuli was the target of two different questions: “Which stimulus was closest to you?” (egocentric question)—“Which stimulus was closest to the bar?” (allocentric question). The specific question was indicated by a word on the screen: “YOU” was for “What stimulus was near to the body”—“BAR” was for “Which stimulus was near to a black bar”. The perceptual judgments (e.g., which was the tallest/shortest object?) were indicated by the word “TALL” on the screen.

After the instructions, the participants were shown each object and asked to name it. This was intended to exclude any difficulties or errors due to naming or visual discrimination in advance. Then, participants received a training session to learn how to use precise buttons to give their response: S for Sphere, C for cube, C for cone, and P for pyramid. The buttons were highlighted in the central area of the keyboard along a vertical axis to minimize lateral effects, while the rest of the keyboard has been hidden. Afterwards, participants underwent a 6-trial training phase during which they were asked to give spatial judgments for dyads not included in the testing phase.

Testing phase and Experimental Design. The experimental session comprised four blocks, one for each spatial judgment and specific dyad (i.e., Egocentric judgment with cube–pyramid dyad; Egocentric judgment with cone–sphere dyad; Allocentric judgment with cube–pyramid dyad; Allocentric judgment with cone–sphere dyad). The order of presentation of the experimental blocks was randomized, as was the order of presentation of the trials within each egocentric/allocentric block. Within the egocentric block, subjects were instructed to answer the egocentric question: “Which object was closest to you”? (i.e., egocentric spatial judgement). Within the allocentric block, subjects were instructed to answer the allocentric question: “Which object was closest to the bar? (i.e., allocentric spatial judgment). In each block, 16 experimental trials were presented regarding egocentric/allocentric spatial judgments and four perceptual judgments as distractors (total experimental trials: 64; total distractors: 16). Distractors were presented randomly in each block, and participants were asked to identify the tallest stimulus. These distractors served to prevent subjects from discerning the real purpose of the experiment.

**Figure 2:**
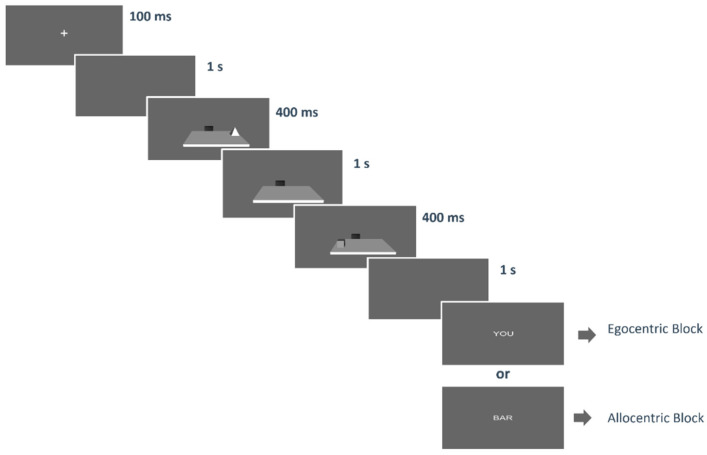
The figure depicts an example of one trial. Each trial started with a fixation cross (100 ms) followed by a blank screen. After 1 s the first object was shown for 400 ms. It could be the egocentric target (i.e., nearest to the participant’s body) or the allocentric target (i.e., nearest to the black bar). Thereafter, only the panel with the black bar was left. Subsequently, the second stimulus was shown for 400 ms: again, this could be the egocentric target or the allocentric target. Finally, the virtual table disappeared, and after a 1 s blank, the word indicating the related question (“you”, “bar”) was presented.

Each experimental trial began with a fixation cross presented on a grey screen for 100 ms, followed by a 1 s blank screen. Then the first object was shown for 400 ms, positioned either closer to the participant’s body (egocentric condition-first) or to the black bar (allocentric condition-first). Next, the second object was shown for 400 ms, positioned either closer to the participant’s body (egocentric condition-second) or to the bar (allocentric condition-second). Subsequently, the virtual table disappeared, and a blank screen was shown for 1 s. Next, a word appeared stating the spatial judgment to be given (YOU’ for the egocentric condition; BAR’ for the allocentric condition) (Figure 2). Perceptual judgments were presented randomly during the spatial task. Participants were instructed to respond as accurately and quickly as possible, with no time limit. Mean accuracy was used to measure performance.

### 2.4. Data Analysis

Mean accuracy for Ego-Allo combined with first-second was computed for each participant. Several ANOVAs and correlation analyses were performed on mean accuracy and scores of the two groups of participants. In detail:

1. A 2 × 2 × 2 ANOVA with “Group” (eAD vs. NC) as a between-subject factor and “Ego-Allo” (i.e., egocentric vs. allocentric judgments) and “Order” (first vs. second) as two within-subject factors was conducted on the mean accuracy of the spatio-temporal Ego-Allo Task;
2. Three one-way ANOVAs with “Groups” as a between-subject factor were carried out on the scores at the FAB, SGORBI, and TMT tests.
3. A 2 × 2 two-way ANOVA with “Groups” as a between-subject factor and “Forward-Backward” as a within-subject factor was performed on the scores at the CORSI test.

For all ANOVAs, the post hoc effects were analyzed using the Bonferroni test, and partial eta-squared (η^2^_p_) was used to report effect sizes.

Additionally, correlation analyses were carried out to investigate the relationship between frontal, attentional, perceptual, and visuo-spatial memory capacities and the ability to process egocentric and allocentric encodings. Specifically:

1. A general correlation analysis on Ego-Allo accuracy as a function of temporal order and scores at FAB, TMT, SGORBI, Corsi Forward, and Backward on the whole sample;
2. The same correlation model described above was applied separately to each group.

## 3. Results

### 3.1. ANOVA on Spatio-Temporal Ego-Allo Task

**Figure 3:**
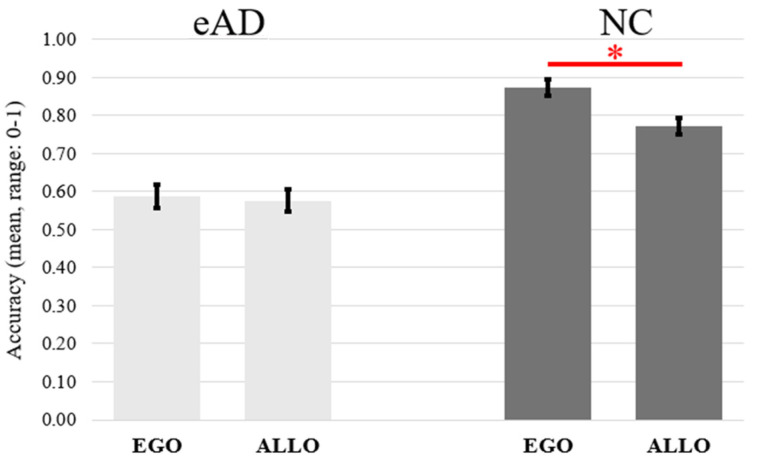
The figure depicts the mean accuracy for egocentric/allocentric judgments as a function of the eAD (early Alzheimer’s disease) and NC (normal control) groups. The small vertical black bars depict the standard error. Significant differences (*p* < 0.05) are indicated by the asterisk.

Results revealed a main effect of Group: F(1,40) = 64.72, *p* < 0.0001, η^2^_p_ = 0.62), with eAD patients (M = 0.58, SE = 0.02; CI +95% = 0.63, −95% = 0.53) performing worse than NC (M = 0.82, SE = 0.02; CI +95% = 0.86, −95% = 0.79). Main effects of “Frame of reference” (F(1,40) = 6.40, *p* < 0.05, η^2^_p_ = 0.14) and “Order” (F(1,40) = 8.74, *p* < 0.05, η^2^_p_ = 0.18) also emerged. Specifically, egocentric (M = 0 0.73, SE = 0.02; CI +95% = 0.69, −95% = 0.77) were more accurate than Allocentric (M = 0.69, SE = 0.02; CI +95% = 0.64, −95% = 0.71) judgments, and judgments were more accurate for first-presented objects (M= 0.73, SE = 0.02; CI +95% = 0.69, −95% = 0.76) than for second-presented ones (M = 0.67, SE = 0.02; CI +95% = 0.64, −95% = 0.71). Moreover, a significant Group x Frames of Reference interaction was found (F(1,40) = 4.16, *p* < 0.05, η^2^_p_ = 0.09) that was due to the higher accuracy of egocentric judgments than allocentric ones in NC (*p* = 0.002) but not in eAD (*p* = 1). Moreover, NC were more accurate than eAD in all comparisons (at least *p* = 0.0001) (see Table 1 and Figure 3).

**Figure 4:**
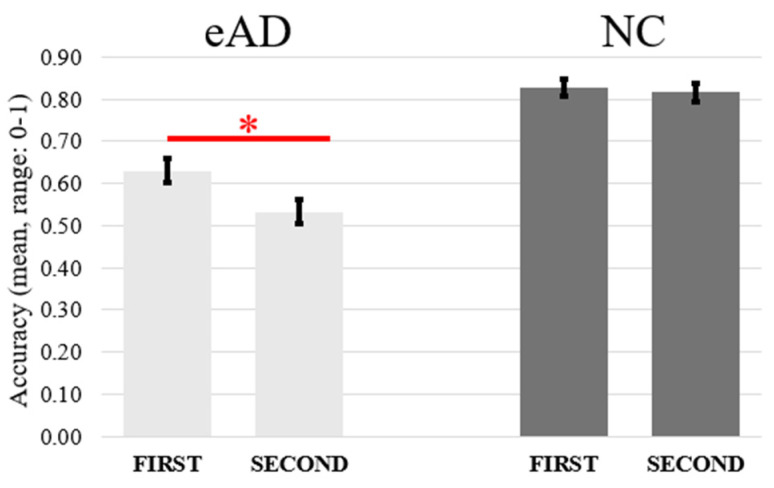
The graph depicts the mean accuracy of first and second egocentric and allocentric judgments as a function of the two eAD and NC groups. The black bars represent the standard error. Significant differences (*p* < 0.05) are indicated by the asterisk.

Additionally, the interaction between Group and Order (F(1,40) = 5.22, *p* < 0.05, η^2^_p_ = 0.12) was due to judgments on first-presented objects being more accurate than second-presented objects in eAD (*p* = 0.02) but not in NC (*p* = 1). Moreover, NC participants outperformed eAD participants in all comparisons (*p* ≤ 0.0001) (see Figure 4 and Table 2 for descriptive statistics).

### 3.2. ANOVA on Dimension Judgment

Although patients with eAD judged the height of objects less accurately (M = 0.74, SE = 0.04; CI +95% = 0.66, −95% = 0.82) compared to NC (M = 0.82, SE = 0.03; CI +95% = 0.76, −95% = 0.88), this difference did not reach statistical significance (F(1,49) = 2.763, *p* = 0.104, η^2^_p_= 0.06).

### 3.3. ANOVAs on Neuropsychological Tests

FAB. Results revealed significant differences between groups: F(1,38) = 28.96, *p* < 0.0001, η^2^_p_ = 0.43. Patients with eAD (M = 11.05, SD = 3.18) performed worse than NC (M = 15.81, SD = 2.35).

TMT. Results revealed significant differences between groups: F(1,38) = 38.27, *p* < 0.0001, η^2^_p_ = 0.50. Patients with eAD (M = 131.71, SD = 74.98) performed worse than NC (M = 36.35, SD = 19.05).

SGORBI. Results revealed significant differences between groups: F(1,38) = 25.45, *p* < 0.0001, η^2^_p_ = 0.40. Patients with eAD (M = 24.27; SD = 5.48) performed worse than NC (M = 30.11, SD = 1.69).

CORSI. Results revealed a significant main effect of “Groups”: F(1,37) = 23.84, *p* < 0.0001, η^2^_p_ = 0.39, with eAD patients performing worse (M = 2.66, SD = 1.25) than NC participants (M = 4.85, SD = 1.37). A significant main effect of “Forward-Backward” was also observed, F(1,37) = 21.80, *p* < 0.0001, η^2^ = 0.37, showing higher span in the Corsi forward than backward. Finally, an interaction between the two factors was found: F(1,37) = 5.22, *p* = 0.03, η^2^_p_ = 0.12. The post hoc test showed that participants with eAD were less accurate in the backward Corsi task (M = 1.58, SD = 1.94) compared to the forward task (M = 3.73, SD = 1.99) (*p* < 0.001). This difference was not observed in NC (backward M = 4.48, SD = 1.80; forward M = 5.26, SD = 0.88). Furthermore, eAD patients performed worse than NC more in the backward (*p* < 0.001) than forward task (*p* = 0.08).

### 3.4. Correlations

#### 3.4.1. Temporal Order Effects on Correlations Between Spatial Judgments and Neuropsychological Tests in the Whole Sample

As shown in Table 3, significant relationships were observed between spatial accuracy and performance across most neuropsychological tests.

Specifically, higher accuracy at the FAB was associated with better performance in both egocentric and allocentric spatial judgments, regardless of whether objects appeared first or second. Similarly, higher scores in the Corsi Forward and Corsi Backward tasks were correlated with higher accuracy in both types of spatial judgments, with slightly stronger correlations observed for egocentric judgments.

Performance on the Sgorbi Test was positively correlated with spatial accuracy, although this relationship was weaker for allocentric judgments of second objects where the correlation did not reach statistical significance (*p* = 0.10).

Consistently, performance on the TMT showed significant negative correlations with spatial judgment accuracy in all conditions. This shows that longer completion times on the TMT were associated with lower accuracy in both egocentric and allocentric judgments.

#### 3.4.2. Temporal Order Effects on Correlations Between Spatial Judgments and Neuropsychological Tests in eAD Group

For egocentric judgments of objects presented first, there were significant positive correlations with performance on the Corsi Forward Test (r = 0.611, *p* = 0.020), the Corsi Backward test (r = 0.84, *p* < 0.001), and the SGORBI (r = 0.53, *p* = 0.049). For egocentric accuracy judgments of objects presented second (EGO-V2-ACC), significant positive correlations were observed with the FAB (r = 0.63, *p* = 0.015) and the Corsi Backward Test (r = 0.58, *p* = 0.029). A negative correlation was observed with the TMT (r = −0.68, *p* = 0.007).

In the case of allocentric judgments of objects presented first, there was a strong positive correlation with the FAB (r = 0.85, *p* < 0.001) and a significant negative correlation with the TMT (r = −0.59, *p* = 0.027). For allocentric judgments of objects presented second, no significant correlations were observed with any of the neuropsychological tests.

#### 3.4.3. Temporal Order Effects on Correlations Between Spatial Judgments and Neuropsychological Tests in NC Group

Egocentric judgments showed a significant positive correlation with the FAB (r = 0.40, *p* = 0.035). For allocentric judgments, two significant correlations were identified: a positive correlation with the FAB (r = 0.38, *p* = 0.047) and a negative correlation with the TMT (r = −0.39, *p* = 0.047).

## 4. Discussion

The present study explored how the temporal order of item presentation influences egocentric and allocentric spatial judgments in individuals with early-stage Alzheimer’s disease (eAD) and normal controls (NC). Participants were required to memorize dyads of 3D geometrical objects presented one at a time on a desk along with a bar. Afterwards, they were required to indicate what stimulus appeared closest to them (egocentric task) and closest to the bar (allocentric task).

Results corroborate prior research by demonstrating that spatial judgments of eAD participants were overall less accurate than those of NC. Notably, the effect sizes of the results overall are in line with previous studies comparing eAD and MCI with NC on similar spatial judgments [24,25]. Moreover, results showed a similar level of impairment in both egocentric and allocentric judgments in eAD patients, suggesting a generalized deficit in spatial processing. Instead, NC were more accurate in egocentric judgments compared to allocentric judgments, consistent with the proposal that egocentric judgments depend on more resilient cognitive processes [36,37].

The spatial difficulties observed in eAD patients are consistent with their general decline across various cognitive domains. In fact, in tests assessing executive functions, such as the Frontal Assessment Battery (FAB), eAD patients performed notably worse than NC, signifying a compromised frontal lobe function. Similarly, tasks that demand working memory and cognitive flexibility, such as the Trail Making Test (TMT), further illustrate these impairments, with eAD patients exhibiting slower reaction times and overall diminished performance. Furthermore, tests assessing sequential spatial memory, like the Corsi Block Tapping Test and Sgorbi Test, respectively, also highlight these cognitive deficits in eAD patients compared to NC. Notably, no significant difference appeared in the simple visual judgment task, and this allows us to exclude that the difficulty of eAD can be ascribed to a general perceptual discrimination limitation. Overall, the higher difficulties in eAD patients compared to the NC highlight impairments in both hippocampal and frontal processes, which are essential for forming and retrieving spatial representations [8,9,10,17,18].

The pattern of results in NC differs from that observed in a previous study with young adults that demonstrated a flexible influence of temporal order on spatial judgments [26]. Specifically, egocentric judgments were more accurate when the object closest to the body appeared first, while allocentric judgments improved when the object nearest to the external reference (the bar) appeared second. This flexibility in spatial processing may reflect an adaptive ability to use different frames of reference in a dynamic context, supported by rapid attention-shifting mechanisms. Unlike younger participants, older adults here did not show a significant temporal order effect. Their spatial judgments were equally accurate for objects presented first or second, regardless of whether the task required egocentric or allocentric judgments. Older adults seem to rely on a processing strategy based on shifting attention from one frame to the other following the task request. Thus, they do not remain anchored on the item that appeared first, but their strategy may be slower, more controlled, and thus less efficient than in young people. This could be due to a reduction in the ability to flexibly shift attention or update working memory with sequential information [38,39,40], which tends to be more difficult in aging [39,40].

However, the key contribution of this study lies in the fact that the effect of temporal order on spatial judgments differed notably between NC and those with eAD. For instance, eAD patients, unlike NC, exhibited a strong influence of temporal order on spatial judgments. Specifically, their performance was significantly worse when making spatial judgments about the object presented second, compared to the first. This temporal order effect was evident across both egocentric and allocentric judgments. While NC still showed a flexible processing strategy, individuals with eAD seemed to struggle more with integrating and updating spatial information over time. This highlights the challenges eAD patients face in dynamically updating their spatial representations, potentially due to deficits in working memory and attentional flexibility [39,40,41,42]. In other words, eAD patients may remain spatially anchored to the first object they encounter because they have difficulty shifting their attention flexibly and updating initial information with new information presented later. The adaptive value of this anchoring strategy would lie in simplifying the task and minimizing the need for demanding cognitive resources. Then, due to the difficulty in forming new memories, the first item remains the only one encoded strongly enough to be recalled.

The results of the spatial judgment task were further clarified by correlating spatial performance with neuropsychological test outcomes. In general, strong associations were found between egocentric/allocentric judgments and executive functions, visuo-spatial working memory, and cognitive flexibility as measured by the FAB, Corsi, and TMT tests [43]. This was true for judgments made on both first- and second-presented objects. However, an interesting exception was found: no significant correlation was observed with the Sgorbi Test and the allocentric judgments of objects presented second, although a positive correlation emerged with egocentric judgments. Consistent with the results about simple visual judgments, this could indicate that the ability of visual discrimination is less involved in the allocentric performance in dynamic contexts.

Interestingly, when analyzing the eAD and NC groups separately, different patterns emerged. eAD individuals showed enhanced dependence on executive functions, working memory, and visual discrimination abilities for egocentric judgments, as demonstrated by correlations with all neuropsychological tests. This reliance suggests that eAD patients may be compensating for their spatial deficits by heavily relying on these cognitive resources. In contrast, NC displayed an association with the executive functions (FAB), possibly indicating a still flexible (though less efficient) use of attentional resources in their spatial performance. This pattern was consistent for both objects presented first and second.

In terms of allocentric spatial judgments, eAD patients showed significant correlations with the FAB and TMT for the first-presented object, but no significant correlations were found for the second-presented object. Conversely, in the NC group, these correlations with allocentric judgments emerged for the second-presented objects rather than the first. This contrasting pattern highlights that eAD patients may struggle to maintain spatial representations over time, particularly under high attentional demands, while healthy controls appear more efficient in their cognitive resource allocation, which enables better performance in sequential spatial tasks.

## 5. Conclusions

Given the importance of spatial symptoms in the genesis of AD, the study aimed to increase our knowledge of factors influencing the ability of patients with eAD to use reference systems to structure spatial information. The focus on the effect of temporal order allowed us to understand not only that conditions of stimulus administration can reveal or mask clinical effects, but also the important link with the capacity to process executive-attentional resources. Indeed, our study highlighted the profound impact of temporal order on spatial memory in individuals with eAD compared to healthy controls. While eAD patients remained anchored to the item presented first, which was more accurate regardless of the frame used, NC were equally accurate with the item that appeared first or second. This suggests a specific difficulty in eAD due to a reduced capacity in the ability to flexibly shift attention and update visuo-spatial memory during dynamic spatial tasks. Instead, healthy controls exhibit a more focused approach, showcasing a better allocation of cognitive resources. The associations between executive functions and spatial judgments emphasize the complex interplay between cognitive capacity and spatial memory in the trajectory from healthy to pathological aging.

Ultimately, our findings emphasize the importance of understanding the cognitive strategies employed by these individuals. By uncovering the compensatory mechanisms at play, we can pave the way for more individualized approaches in supporting the cognitive needs of patients affected by Alzheimer’s disease. The journey toward better navigational assistance and cognitive support for eAD patients begins with continued exploration and innovation in our understanding of their unique cognitive landscape.

### 5.1. Summary

Alzheimer’s disease leads to early and pronounced impairments in spatial memory and navigation. These deficits are linked to damage in the HP-EC network, impacting place and grid cells responsible for spatial mapping. Allocentric memory is affected first, diminishing the ability to navigate based on external cues and causing a reliance on egocentric strategies [44]. Prior studies highlight how these changes distinguish AD patients from healthy controls and suggest that allocentric deficits, detectable through VR and imaging studies, could serve as early indicators of AD. This research underscores the importance of spatial memory assessment in diagnosing and tracking AD, particularly through tasks that test both egocentric and allocentric abilities in dynamic conditions [45]. This would make it possible to define intervention strategies aimed at training spatial memory capacities through tasks close to the demands of everyday life, with beneficial implications for both AD patients and caregivers.

### 5.2. Limitations of the Study

Two main limitations of the present study must be emphasized. First, only 14 patients with eAD were recruited in this study; a larger sample size would allow for more robust results and conclusions as well as better generalizability of the findings. Second, the current study does not allow for the possibility to assess changes in egocentric and allocentric spatial representation over time in eAD patients. Therefore, further research is needed for more solid conclusions and to gather information on changes in spatial memory abilities over time in elderly people diagnosed with eAD.

## Figures and Tables

**Table 1:** Mean accuracy (and standard error, SE) for egocentric and allocentric judgments in eAD and NC groups, with their respective ±95% confidence intervals.

		eAD		NC
	EGO	ALLO	EGO	ALLO
MEAN	0.59	0.58	0.87	0.77
SE	0.03	0.03	0.02	0.02
−95	0.53	0.51	0.83	0.73
+95	0.65	0.64	0.92	0.82

**Table 2:** Mean accuracy (and standard error, SE) for judgments on the first-presented and second-presented objects in the eAD and NC groups, with their respective ±95% confidence intervals.

		eAD		NC
	FIRST	SECOND	FIRST	SECOND
MEAN	0.63	0.53	0.83	0.82
SE	0.03	0.03	0.02	0.02
−95	0.57	0.47	0.79	0.77
+95	0.69	0.59	0.87	0.86

**Table 3:** Correlations Between Neuropsychological Tests and Spatial Judgments (Egocentric and Allocentric Accuracy) according to the order of presentation of the objects (1 = first; 2 = second).

	EGO-1	EGO-2	ALLO-1	ALLO-2
FAB	r = 0.69, *p* < 0.001	r = 0.71, *p* < 0.001	r = 0.61, *p* < 0.001	r = 0.60, *p* < 0.001
Corsi Forward	r = 0.62, *p* < 0.001	r = 0.56, *p* < 0.001	r = 0.49, *p* = 0.001	r = 0.45, *p* = 0.004
Corsi Backward	r = 0.67, *p* < 0.001	r = 0.64, *p* < 0.001	r = 0.46, *p* = 0.003	r = 0.35, *p* = 0.025
SGORBI	r = 0.65, *p* < 0.001	r = 0.69, *p* < 0.001	r = 0.40, *p* = 0.010	r = 0.27, *p* = 0.098
TMT	r = −0.68, *p* < 0.001	r = −0.81, *p* < 0.001	r = −0.59, *p* < 0.001	r = −0.48, *p* = 0.002

## Data Availability

The data presented in this study is available on request from the corresponding author.
